# Spontaneous Local Gamma Oscillation Selectively Enhances Neural Network Responsiveness

**DOI:** 10.1371/journal.pcbi.1000342

**Published:** 2009-04-03

**Authors:** Se-Bum Paik, Tribhawan Kumar, Donald A. Glaser

**Affiliations:** 1 Department of Physics, University of California Berkeley, Berkeley, California, United States of America; 2 Department of Molecular & Cell Biology, University of California Berkeley, Berkeley, California, United States of America; University College London, United Kingdom

## Abstract

Synchronized oscillation is very commonly observed in many neuronal systems and might play an important role in the response properties of the system. We have studied how the spontaneous oscillatory activity affects the responsiveness of a neuronal network, using a neural network model of the visual cortex built from Hodgkin-Huxley type excitatory (E-) and inhibitory (I-) neurons. When the isotropic local E-I and I-E synaptic connections were sufficiently strong, the network commonly generated gamma frequency oscillatory firing patterns in response to random feed-forward (FF) input spikes. This spontaneous oscillatory network activity injects a periodic local current that could amplify a weak synaptic input and enhance the network's responsiveness. When E-E connections were added, we found that the strength of oscillation can be modulated by varying the FF input strength without any changes in single neuron properties or interneuron connectivity. The response modulation is proportional to the oscillation strength, which leads to self-regulation such that the cortical network selectively amplifies various FF inputs according to its strength, without requiring any adaptation mechanism. We show that this selective cortical amplification is controlled by E-E cell interactions. We also found that this response amplification is spatially localized, which suggests that the responsiveness modulation may also be spatially selective. This suggests a generalized mechanism by which neural oscillatory activity can enhance the selectivity of a neural network to FF inputs.

## Author Summary

In the nervous system, information is delivered and processed digitally via voltage spikes transmitted between cells. A neural system is characterized by its input/output spike signal patterns. Generally, a network of neurons shows a very different response pattern than that of a single neuron. In some cases, a neural network generates interesting population activities, such as synchronized oscillations, which are thought to modulate the response properties of the network. However, the exact role of these neural oscillations is unknown. We investigated the relationship between the oscillatory activity and the response modulation in neural networks using computational simulation modeling. We found that the response of the system is significantly modified by the oscillations in the network. In particular, the responsiveness to weak inputs is remarkably enhanced. This suggests that the oscillation can differentially amplify sensory information depending on the input signal conditions. We conclude that a neural network can dynamically modify its response properties by the selective amplification of sensory signals due to oscillation activity, which may explain some experimental observations and help us to better understand neural systems.

## Introduction

Understanding the responsiveness of a cortical neural network is a fundamental requirement for any study of sensory information processing in the brain. Several experiments show that various factors can affect the neuronal response property and information flow in nervous systems: In the primary visual cortex, spiking responses of neurons can be enhanced by slow cortical oscillation [1]. The spike transfer function of thalamo-cortical neurons is modulated by noisy synaptic background activity [2]. Gain of neuronal responses is modulated by background synaptic input [3]. Even at the single cell level, cellular responsiveness is significantly influenced by the presence of voltage fluctuations [4]. It was shown recently that neuronal oscillations can increase response gain and decrease reaction time as a mechanism of attention selection [5].

Cortical neurons commonly show synchronous or oscillatory patterns of activity, which is thought to be important for cortical functions of information flow [6]. In particular, synchronous gamma frequency oscillations (30∼70 Hz) have been observed in various neural circuits [7],[8],[9], and they are thought to provide a temporal structure for information processing in the brain [10]. This gamma-band synchronization can be generated within local networks by coupling between GABAergic I- (inhibitory) interneurons and E- (excitatory) neurons [11],[12],[13],[14], and is related to cognitive functions [15],[16], and information delivery [15]. This population activity also has been studied in numerical simulations and mathematical models [12],[17],[18],[19].

Previous analyses have shown that cortical oscillations are generated in networks with appropriate connectivity and can be correlated with the firing phases of E- and I- neurons, but the effect of these oscillations on the neural network responsiveness to external inputs remains elusive. In this research, using a large network model of Hodgkin-Huxley type E- and I- neurons, we study how spontaneous cortical oscillation - particularly in the gamma frequency band - modulates the response property of a neural network. We examine the cortical responsiveness to external FF inputs at the single-spike level because the input-output response function for a single input spike is a fundamental feature of neural networks for information processing. A recent study emphasizing the importance of single spike level analysis showed that a significant amount of visual information can be delivered by the very first spike emitted by a neural population [20].

We found that spontaneous cortical oscillation activity noticeably changed the cortical input-output response function. For example, weak inputs that are normally missed in the responses of single neurons, were significantly enhanced by cortical oscillations in the network. This response modulation was similar to the observed effect of the membrane potential oscillation reported in a previous experimental study [21]. More importantly, we found that this cortical response modulation by the oscillation activity was controlled by external feed-forward (FF) input strength variation. This means that the cortical network can self-regulate by differentially amplifying its FF inputs according to their strength, without physiological changes in single neuron properties or structural modulation of interneuron connectivity. We show that this ‘differential’ amplification results from the modulation of gamma oscillation by cortical E-E neuron interaction. We suggest that this is an important example in which the modulation of gamma oscillation by cortical E-E interactions [12] can significantly change the population responsiveness. We also found that this cortical amplification effect was restricted spatially to an ‘oscillation active’ region, which enables the spatially-selective tuning of responsiveness to given FF input. We find that this spatial localization is determined by the range of anatomical interneuron connectivity. This is consistent with recent experimental findings concerning the effects of cortical oscillations [1],[5],[22], and points to aspects of this oscillation which effectively enhance the response selectivity of a neural network to FF inputs.

## Results

### Cortical Network Model

We examined a variety of neural network activity patterns produced under different conditions. We used a cortical network model in which E- and I- neurons interact with each other via lateral synaptic connections. We constructed isotropic local cortical connections, using physiological and earlier modeling data [23],[24]. We varied the strength of each type of cortical interaction (E-E, E-I, I-E and I-I) in order to study different cortical connectivity conditions. Artificially generated random spikes were injected into the cells in the center area (diameter∼450 µm, ∼500 neurons: E- 377, I- 113) of this network model (1 mm by 1 mm, consisting of ∼3300 neurons: E- 2500, I- 841) to simulate localized FF spike input to the network. The actual spike pattern for each neuron was generated by a Poisson process with constant mean firing rate, and FF input strength (The amount of intracellular conductance fluctuation caused by a single FF input spike) was varied within the range 5∼100 µS/cm^2^, as a control parameter. By performing many simulations of different cortical parameters with FF input rates in the range 5∼40 spike/s, we observed several different types of cortical activity patterns.

### Cortical E- and I-Cells Activity: Generation of Gamma Oscillation

**Figure 1:**
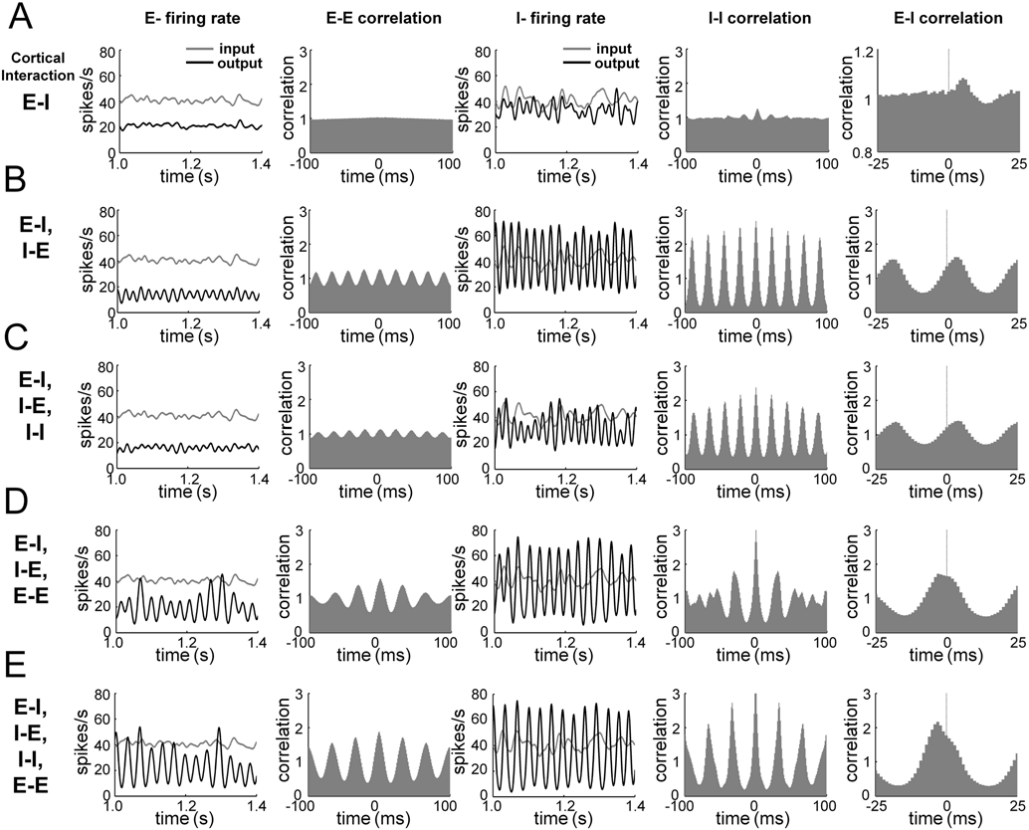
Generation and modulation of spontaneous cortical oscillation. Instantaneous FF input rate and cortical output firing rate (column 1 and 3). Spikes in each neuron were smoothed with a Gaussian function (σ = 5 ms) and averaged across neurons. Cross-correlogram of cortical output spikes (column 2, 4 and 5). This indicates the probability that cortical spikes from different neurons are separated in time by the indicated x value. It was normalized so that the uncorrelated state is set to unity. (A) E- cells synchronize I- cells by cortical E-I connection. I-I correlogram has a small peak at t = 0, and E-I correlogram shows that I- cells fire ∼5 ms after E- cells fire. (B) The cortical gamma frequency rhythm is generated by E-I and I-E connections. I- cells fire at higher average rate than E- cells, and E- and I- cells fire with a fixed time delay (∼5 ms) and a fixed firing phase difference (∼70-degrees) in each cycle. (C) The I-I interaction reduces the firing rate of I- cells, but does not change the E-I firing phase (D) The E-E interaction significantly changes the relative phase between the firing patterns of E- and I- cells. E- and I- cell spikes are almost in phase. (E) With all four types of cortical connections, the difference between the firing rates of E- and I- cells diminished, and the cortical oscillation frequency was lower than (B), (C). E- and I- cells fire almost in phase, or I- cells fire slightly before E- cells fire.

Gamma oscillation can be generated by interactions among E- and I- cells: The E- cells synchronize the I- cells, and the gamma-modulated I- cells drive E- cells to generate gamma frequency rhythms in the network [11],[14],[25]. Our simulations agreed with earlier studies that gamma oscillations are generated by applying E-I and I-E cortical connections; I- cells were synchronized by E-I connections first (Figure 1A), then I-E connections generated gamma rhythm in E-cells via periodic inhibitory activity (Figure 1B) as in the PING model [11],[14]. I- cells fire at higher rate than E- cells, just as fast-spiking cells fire at higher rates than regular-spiking cells [10]. The relative firing phase of E- and I- cell (Figure 1A and 1B) also showed a similar phase relation as reported in the previous experiments [25],[26],[27]. E- cells fire 3∼5 ms before I- cells fire, with an approximately 70-degree phase difference in a gamma cycle.

We extend the previous studies by also explicitly considering E-E and I-I cortical connections. In ref. [25], Morita et al. showed that gamma- modulated cortical excitation increases the firing rate of E- cells and decreases the E-I firing phase difference. Based on these observations, they predicted that the gamma- modulated E-E cell interaction will suppress the cortical oscillation. In this study, we found that the gamma- modulated E-E coupling does not extinguish the cortical oscillation. When E-E connections were turned on, the firing rate of E- cells was increased and the E-I phase difference was diminished (Figure 1D and 1E), as shown in ref. 25. But these changes did not actually suppress the cortical oscillation. Instead, they caused a transition of operating ‘mode’ such that the oscillation frequency dropped to a low gamma or near beta rhythm [28]. This transition could not be observed using the methods reported in ref 25, because they have only ‘static’ data of pre- and post- synaptic activities to examine ‘static’ equilibrium conditions.

The approximately zero E-I firing phase difference is an important feature of E-E coupling, and is distinguishable from that of the case involving no or little E-E coupling (∼70-degrees is usually observed in experiments [26],[27]), so we call the former situation ‘E-E interaction modulated’ E-I phase, in contrast to the ‘normal’ gamma oscillation E-I phase. We will analyze below how this phase modulation results from E-E coupling.

The difference between the firing rates of E- and I- cells is also diminished to some degree by E-E interactions, and the population oscillation frequency is also lowered [28],[29]. We maintain this non-zero E-E interaction throughout the following simulations. We also allow I-I connections, which reduce the firing rate of I- cells to some extent (Figure 1C and 1D). In this study, we do not examine the role of I-I connection in detail, and the I-I connection strength was not varied.

### Control of Spontaneous Cortical Network Activity Strength

**Figure 2:**
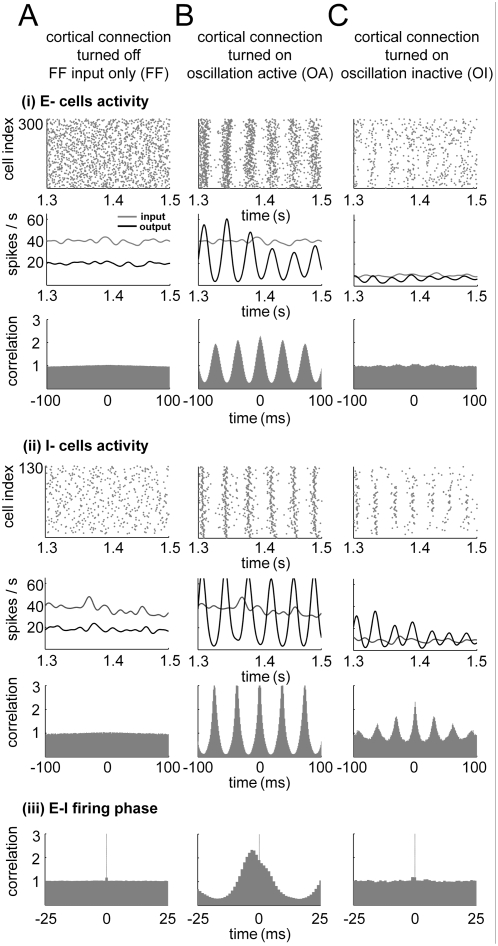
Various cortical activity states. Raster plot of cortical output spikes, instantaneous FF input and cortical output firing rate, and cross-correlogram of cortical output spikes. (A) (i), (ii) When cortical synaptic connections are turned off, there is no correlated cortical activity. The output firing rate directly follows the FF input rate pattern. (iii) The E-I firing phase shows no correlation. (B) (i), (ii) When cortical connections are turned on, both E- and I- network neurons show oscillation patterns for a wide range of FF input and cortical parameters. This spike raster plot shows grating-like patterns, and the output firing rate oscillates with the gamma band frequency. The cross-correlogram pattern also shows a clear oscillation pattern. (iii) In this case, the E- and I- cells fire with little phase difference. This E-I firing pattern is different from normal gamma oscillation in the E-I phase, indicating that the contribution of the E-E interaction is significant in this case (see Figure 1E). (C) (i), (ii) The oscillation-inactive state can be achieved simply by lowering the FF input rate. The E-E cross-correlogram shows hardly any oscillation pattern, even though cortical connectivity is kept the same as in (B) and I- cells show oscillation patterns. The FF Input rate was 40 spikes/s for (A) and (B), and 10 spikes/s for (C). (iii) The E-I firing phase shows no clear correlation as in (A) (iii).

When the cortical interactions (E-E, E-I, I-E and I-I) are completely turned off and each neuron is driven by FF inputs only, there is no correlated behavior in the network and the average network firing rate simply follows the instantaneous input firing rate (Figure 2A, FF). On the other hand, when cortical interactions (E-E, E-I, I-E and I-I) are introduced, the network exhibits some synchronized patterns depending on FF input and cortical connectivity parameters. For example, with a moderate FF input strength (∼35 µS/cm^2^) and rate (∼40 spikes/s), the neural population shows an oscillation pattern in its firing rate for a broad range of cortical connectivity parameters. The cross-correlogram among cortical spikes shows a clear oscillatory pattern for both E- and I- cells in this case (Figure 2B, OA). Generally, the amplitude of the instantaneous output firing rate of the neural population depends on the FF input firing rate. The frequency of the oscillation is somewhat affected by the FF input strength and cortical connectivity parameters, but the oscillation frequencies are mostly within the gamma band range (25∼50 Hz), as in earlier experimental observations and theoretical models [8],[9],[30],[31]. For some parameter range of cortical connections, this gamma oscillation can be very small. When the FF input spike rate was low (∼10 spikes/s) with the other parameters unchanged, the oscillating firing pattern in E- cells became barely detectable even though the oscillation in I- cells was maintained to some extent (Figure 2C, OI). We examined the phase of the firing pattern of E-I cells in three cases (FF, OA, and OI). The E-I spike correlogram in the OA network showed that the effect of interactions among E-E cells is significant because the E- and I- cells fire with little phase difference (Figure 2B (iii)), similar to Figure 1D and 1E (‘E-E interaction modulated’ E-I phase), while the other two cases (FF and OI) showed no clear phase relation (Figure 2A (iii) and 2C (iii)). Since OA and OI networks have the same parameters except for the FF input firing rate, we conclude that FF input firing rate is a crucial factor in determining E-E interaction strength.

When E-E cortical connections are very strong, extremely sharp cortical spike synchronization is generated, resulting in spatially propagating waveform patterns [32],[33],[34]. The amplitude of the instantaneous output firing rate was almost constant, independent of FF input rate. In this case, the neural response was not controlled very much by FF inputs at each moment but mostly by the spatial cortical bursting pattern. We observed that the frequency of this periodically propagating pattern is in the beta oscillation range induced by E-E cortical interactions [28]. We do not examine the generation or the effects of periodically propagating patterns any further here. In the following simulations, we chose cortical conditions such that the system didn't enter this phase for the FF input strengths tested.

To examine the responsiveness of the neural network, we chose a range of parameters that provided moderate and stable oscillatory behavior on application of FF input spikes. We tested this condition using sinusoidal time-varying input rates with a peak amplitude range of 0∼60 spikes/s and peak frequency of 5∼10 Hz. The network rapidly and reliably restored its oscillating state whenever the FF input firing rate became greater than some level (∼20 spikes/s), and the oscillations diminished significantly and very rapidly when the input rate fell below that level. Throughout this research, we did not change any individual neuronal property (e.g. ionic channel parameters).

### Network Activity Increases the Cortical Response to Weak FF Inputs and Modifies the Response Function

We compared the neural responsiveness for the following three states of network activity: (i) network with no cortical connectivity (and no spontaneous network activity) (FF), (ii) laterally connected cortical network with spontaneous oscillation activity (OA) and (iii) the same network as in (ii) but with little or no oscillation activity (OI).

We generated random FF spike inputs by a Poisson process and provided this input to the center area (diameter∼450 µm) of the 1 mm by 1 mm network. All the response properties were measured within this small center area in order to avoid finite size effects from the network boundary. Neurons outside this area received no FF input. FF input strength was varied from 5 to 100 µS/cm^2^, and FF spike rate was kept constant at 40 spikes/s for (i) and (ii). For (iii) the oscillation inactive case, the FF input rate was lowered to 10 spikes/s in order to maintain minimal oscillations while still providing enough input spikes to allow measurement of the network responsiveness.

**Figure 3:**
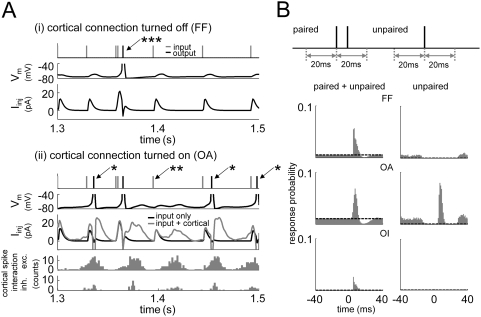
Responsiveness to weak FF inputs is enhanced by spontaneous cortical gamma frequency oscillation. (A) (i) Cortical connections are turned off (FF). Neurons receive only FF inputs. Each weak (<30 µS/cm^2^) FF input raises some current and voltage fluctuation but cannot cause a cortical output spike unless two or more inputs are closely paired (***). (ii) Cortical connections are turned on (OA). In the presence of cortical gamma oscillation, each neuron receives cortical spikes from nearby neurons. Since cortical activity has the gamma frequency oscillation pattern, each neuron is provided with a periodic current fluctuation. This cortical current amplifies weak FF inputs to drive output spikes (*). This response enhancement depends on the phase of oscillation, so a FF input at an oscillation node is not amplified (**). (B) The response probability of entire (paired+unpaired) and single (unpaired) FF inputs (weak input strength ∼25 µS/cm^2^) for different cortical activity states. In both cases (paired and unpaired), input spikes were chosen only if there were no other spikes within 20 ms before each input. Paired inputs have another input within 20 ms after each input. Cortical output spikes were counted as is their relative timing to each input spike were 0 ms). All three cases show a non-zero response peak for the entire input. For unpaired input, only the OA network can respond. In each correlogram, response probability was normalized by the number of proper (entire or unpaired) FF input spikes. For the responsiveness calculation (Figure 3A), each peak area was measured above background activity level (dashed line). Background activities were measured within a window from −10 ms to 0 ms.

**Figure 4:**
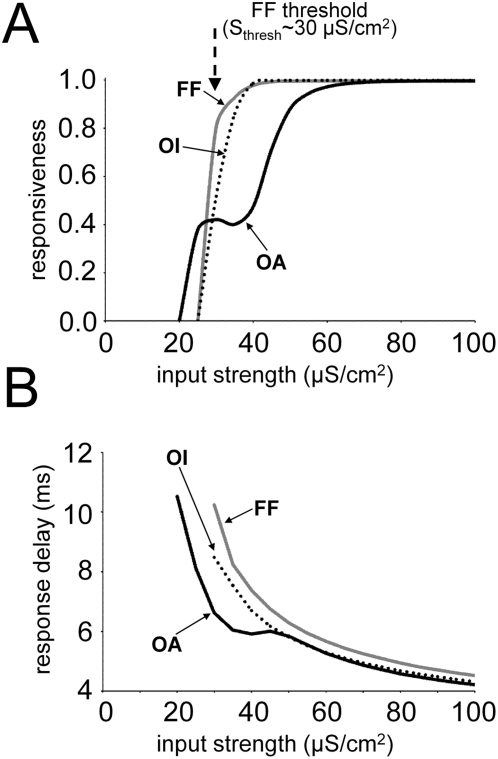
Responsiveness and response delay of each cortical state. (A) The FF network has a step-like shape response function, with a threshold value of ∼30 µS/cm^2^. The OA network shows a more gradual change in its response function with a plateau near the FF response threshold. Its responsiveness for weak FF inputs is much stronger than for the other two cases. The OI network shows little difference in its response function from that of the FF network. (B) The cortical response to a single FF input spike is fastest in the OA network for all input strengths. For weak FF inputs, the average response delay is much shorter in the OA network than in other network states. As the FF input strength increases, the difference between OA and OI networks becomes smaller. The FF network always shows the largest delay time, even for very strong FF inputs.

Figure 3A shows FF input and cortical output spike trains, the membrane voltage, and the internal current fluctuation of a sample neuron with and without network oscillation activity. Each FF input spike induces a synaptic conductance fluctuation in a cortical neuron and the corresponding intracellular current fluctuation. When cortical interactions are turned off, the response of each neuron depends only on the direct FF input (FF network, Figure 3A (i)). When the FF input strength is weak (25 µS/cm^2^), a single spike input could not produce a large enough current fluctuation to generate an output spike. Only when two or more inputs are temporally paired within a short time interval (<∼10 ms), can the conductance fluctuations from each spike overlap to generate an output spike (Figure 3A (i) ***), as found in the measurements of correlated inputs in a previously reported experiment [35]. In this case, there is a ‘threshold FF input strength (S_thresh_)’ that determines whether each ‘single’ FF input spike can generate an output spike or not. We calculated the responsiveness of network neurons, using a cross-correlation method (Figure 3B) [35],[36]. The responsiveness of the cortical network was defined as (net peak integral)/(number of FF input spikes), where net peak integral is the total area of the maximum response peak above the background activity in each cross-correlogram. Measuring this quantity at each FF input strength produces the generalized response function of the network (Figure 4A). Since any subthreshold single input produces no response when cortical connections are turned off (Figure 3B unpaired), the response to a single FF input spike is like a step function, similar to the measured thalamo-cortical transfer function in the absence of noisy background activity [2], providing the cell with a simple spike relay capability with limited encoding abilities. When the cortical connections are turned on, each neuron receives cortical synaptic inputs from other neurons as well as FF inputs. In the presence of the spontaneous network activity of synchronized oscillating patterns, the cortical spike inputs that each neuron receives are also oscillating (Figure 3A (ii)). The current fluctuation due to cortical interactions is added to that by FF inputs, and as a result, a single-spike sub-threshold FF input can produce an output spike response with the help of this additional cortical activity (Figure 3A (ii) *). This input amplification depends on the phase of the cortical activity. When FF input timing is out of phase with the cortical oscillation, it fails to produce an output spike for lack of additional cortical current (Figure 3A (ii) **) just as in the FF network. The general response function of the network to a single ‘unpaired’ input spike is plotted in (Figure 4A), as a function of FF input strength. Different from the step-like FF case, the slope of the response function changes more gradually, with a plateau near the FF threshold input strength (S_thresh_∼30 µS/cm^2^). This broader and more gradual change of response function can provide richer encoding capabilities [2].

Next, we examine how responsiveness changes when the oscillation is inactive while the connectivity of neural population is kept the same. We lower the FF input rate to 10 spike/s, so that the spontaneous oscillation is almost absent. All the other parameters including cortical connectivity are kept the same so that each synaptic interaction via spike delivery in the network can give the same contribution as before. This time, neurons do not exhibit enhanced responsiveness for weak inputs; each neuron still experiences some conductance change by cortical interaction, but its contribution is negligible. The network response character is similar to that of the FF network (Figure 3A). Any ‘unpaired’ weak inputs cannot generate a cortical response, losing its information. We found the response function of neurons is almost the same as that of the FF network (Figure 4A) when the cortical connections are turned off.

### Oscillation Strength is Self-Modifiable: Cortical Modulation Is Controlled by FF Input Strengths

**Figure 5:**
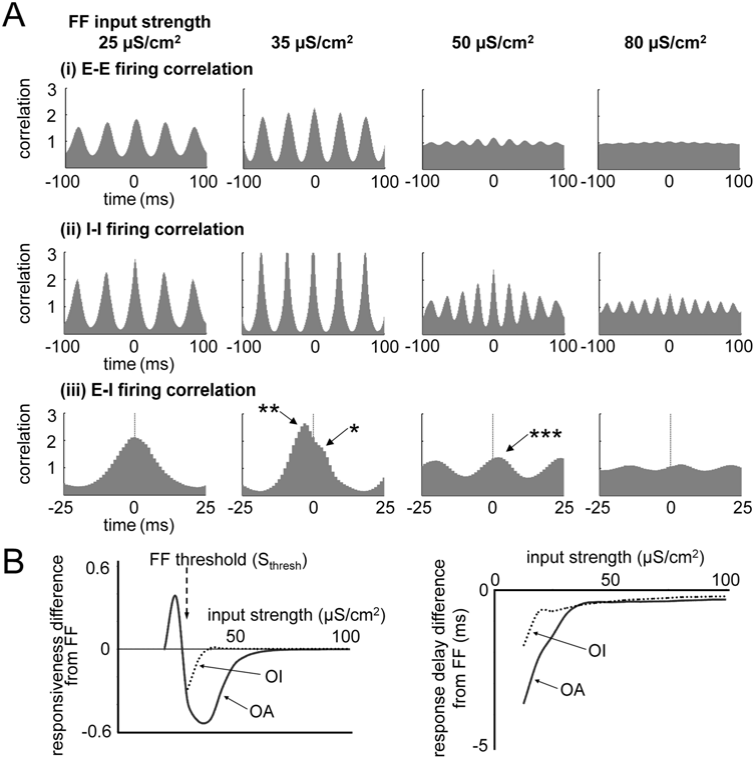
Modification of oscillation strength by various FF input strengths. (A) Cross-correlograms of cortical spikes. (i), (ii) In the OA network, the relative strength of gamma oscillation changes according to the FF input strength. The oscillation is strongest when FF input strength is 35∼40 µS/cm^2^, and gradually diminishes as input strength increases. (iii) The E-I firing pattern shows that cortical oscillation is significantly modulated by the E-E interaction, and two different peaks coexist (‘spike doublets’, * and **). For a stronger FF input (50 µS/cm^2^), the E-I firing pattern shows only the normal gamma phase feature (***). Cortical oscillation is almost disappears for very strong FF input (80 µS/cm^2^). (B) The responsiveness difference between the OA network and the FF network changes from positive (for weak FF inputs<S_thresh_) to negative (for moderate FF inputs>S_thresh_), and becomes zero (for strong FF inputs). Its absolute value (or cortical response modulation) is almost proportional to the strength of the gamma oscillation at each input strength (except at the FF response threshold, ∼30 µS/cm^2^, where the difference changes from positive to negative). Also the difference in response delay between the OA network and the FF network is proportional to the strength of the gamma oscillation, which is controlled by the FF input strength.

In Figure 4A, the absolute difference in responsiveness between the oscillatory network and the FF network is large when FF input strength is weak (near the FF response threshold, S_input_∼30 µS/cm^2^). This difference becomes smaller as the FF input strength increases. Finally the two response functions become equal at very strong inputs (S_input_>80 µS/cm^2^). In other words, the cortical amplification due to oscillation activity is large for weak FF inputs, and becomes insignificant as inputs become strong. We examined how this cortical response modulation is related to the change of oscillation strength. Figure 5A shows values of the network oscillation as a function of FF input strength. For weak FF inputs (S_input_ = 25, 35 µS/cm^2^), the cortical oscillation is strong but it becomes weaker for a medium input strength (S_input_ = 50 µS/cm^2^), and almost disappears for a strong input (S_input_ = 80 µS/cm^2^). This change in the oscillation strength is almost proportional to the extent of the cortical response modulation which can be roughly defined as the difference between the response function and that of the FF network (Figure 5B).

**Figure 6:**
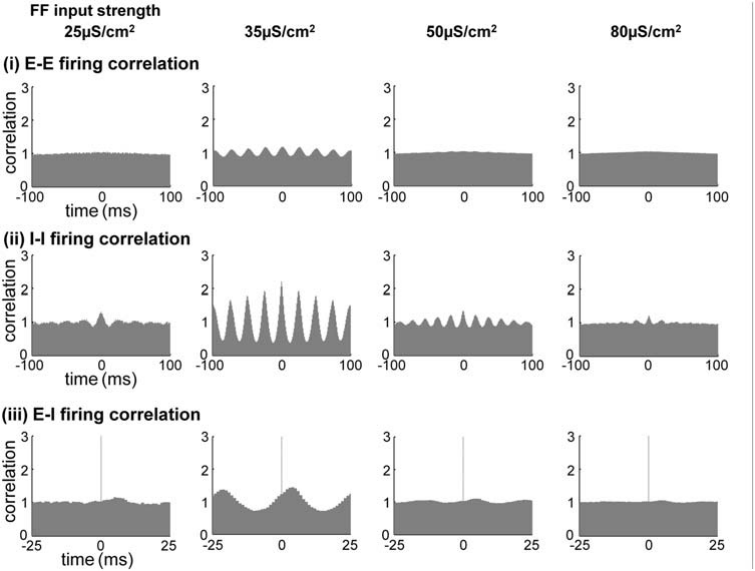
Oscillation modulation without E-E interaction. (i) (ii) The cortical oscillation strength is weaker, and far less modified by the FF input, compared with Figure 5A. The oscillation pattern in E- cells disappears for both weak and strong FF inputs. (iii) The relative E-I firing phase does not change for weak inputs in this case, unlike that shown in Figure 5A (iii).

As shown by examining the E-I firing phase pattern (Figure 5A (iii)), the oscillation modulation is significantly affected by the E-E cell interaction. When the FF input is strong, the E-I spike correlogram shows ‘normal’ E-I phase difference of the gamma oscillation (Figure 5A ***). On the other hand, when the FF input is weak, the relative E-I firing phase is near zero (‘E-E interaction modulated’), showing that the network is affected by strong E-E interaction (Figure 5A (iii), S_input_ = 25, 35 µS/cm^2^). For some range of FF input strength, two different peaks coexist in the E-I correlerogram (Figure 5A * and **). Whenever the E-I firing phase is significantly affected by the E-E interaction (∼70-degrees→∼0-degrees), the cortical oscillation becomes strong and the responsiveness of the network to weak FF input is enhanced. When the E-E connections were turned off (Figure 6), there was no E-I firing phase modulation (Figure 6 (iii)), and the cortical oscillation was far less affected by the FF input strength (Figure 6 (i)). For weak FF input, the cortical oscillation almost disappeared (Figure 6 (i) input strength 25 µS/cm^2^) and consequently the responsiveness did not exhibit any enhancement.

**Figure 7:**
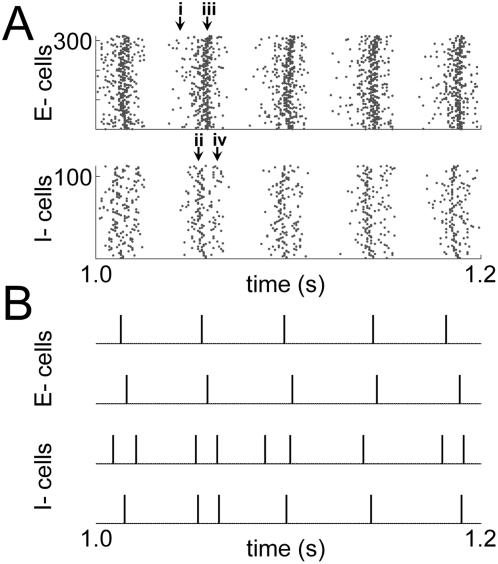
The E-I firing phase modulation by E-E coupling. Raster plot of E- and I- cells spikes (A) and spike trains in sample E- and I- cells (B). Only excitatory connections (E-E and E-I) are turned on, while inhibitory connections (I-E and I-I) are turned off. (A) At first, a small number of E- cells fire by FF input (i). These E- spikes stimulate nearby E- and I- cells by E-E and E-I connections respectively. Since the E-I connection is stronger than the E-E connection in this model, I- cells fire (ii) before E- cells fire (iii) in this cortical drive. The E- cells firing by means of the E-E interactions continues for a while, due to the self-feedback in the E-E interaction loop. As a result of this ‘lagged’ synchronization, the oscillation frequency is reduced. This duration of excitation causes the second firing of I- cells (iv) to make a ‘spike doublet’. The interval between the two spikes in a spike doublet is determined by the refractory period of I- cells. The first spike of a doublet forms the ‘E-E interaction modulated phase’ in the E-I firing phase, while the second spike forms the ‘normal’ gamma E-I phase. (B) I- cells occasionally produce ‘spike doublets’. The first I- spike in a doublet usually fires before nearby E- cells spike, while the second I- spike usually follows the E- spike. If inhibitory connections (I-E and I-I) are turned on, E- cells fire less than once in each cycle, and I- spike doublets appear less frequently.

We examined how the E-I firing phase is modulated from ∼70-degrees to ∼0-degrees by the excitatory interactions in the E-E couplings. We turned on only excitatory cortical connections (E-E and E-I) and measured the firing patterns of E- and I- cells (Figure 7). In this condition, the network generated the periodically propagating waves near beta rhythm that we observed in the earlier part of this study (when E-E connections were relatively stronger than other types of connections). In each wave cycle, a small number of E- cells fired due to the FF inputs (Figure 7A i). Then they excited nearby E- and I- cells through the E-E and the E-I connections. The stimulated E- and I- cells fired almost simultaneously, or I- cells fired slightly before E- cells fire (Figure 7A ii and iii) because E-I connections are stronger than E-E connections. The firing of the E- cells continued for a while due to the ‘chain reaction’ of E-E couplings and the I- cells occasionally produced ‘spike doublets’ by this sustained excitation (Figure 7A iv and 7B). These inhibitory spike doublets are frequently observed during long range synchronization processes in neural networks [9], usually along with the ‘delayed’ excitation from distant cells. In our study the E→E→I route could provide the ‘delayed’ or ‘sustained’ excitation. As a result of these E- and I- firing patterns, the E-I firing phase was ∼0-degrees on average. When the average excitation to each I- cell is strong enough to produce I- spike doublets, the E-I phase correlogram has two peaks around 0-degrees (Figure 5A * and **). If the excitation to I- cells is not enough, or the inhibition of E- cells is strong so that the sustained E- cell activity cannot drive I- cells to produce the second spike of spike doublets, the E-I phase correlogram does not show clear peak separation (Figure 5A (iii) input strength 25 µS/cm^2^). In any case, the average E-I phase difference is close to 0-degrees, clearly different from ∼70-degrees ‘normal’ gamma phase distribution.

The cortical response modification caused by this oscillation can be explained as an effect of membrane potential oscillations [21]. In addition, the modulation of the E-E interaction by the FF input strength can be understood as follows: as shown in Figure 3A, when the FF input strength is weak, each E- neuron can respond to individual FF input only with the help of the collective cortical activity of E- cells whose contribution is intrinsically periodic (oscillatory). The probability of a cortical response to an FF input spike strongly depends on the phase of oscillation of the E- cells (Figure 3A * and **), and this dependence is significantly strengthened by the E-E coupling loop. In the beginning of a gamma oscillation cycle, only a small number of E- cells fire together, but they trigger an E-E coupling loop which drives more E- cells to fire simultaneously. As a result, the peak firing rate of E- cells in a gamma cycle is quite high, causing much higher spiking probability near the oscillation peak. Therefore, (i) the cortical responsiveness is dependent on the oscillation phase, and (ii) the gain or the cortical amplification is proportional to the oscillation strength.

When an FF input is strong enough to produce an output spike in each E- cell, there is no significant dependence of the E- cell response on the periodic cortical activity and E-E couplings, and each individual E- cell responds directly to its FF input pattern, independently of the network activity. Since the FF input spike train was generated by a random Poisson process, the cortical response pattern is also random and not oscillatory. Although the average output firing rate generally increases with increasing FF input strength, the cortical response modulation actually decreases. The oscillations are depressed by increasing the FF input strength while keeping the FF input rate constant. The average number of FF input spikes does not decrease but the oscillation is depressed, and the response function converges to the FF result. Although cortical oscillation is initially established by the E-I and the I-E interactions, the E-E cell interaction is crucial for responsiveness modulation because its strength strongly depends on the FF input strength. Since the cortical response modulation is controlled by the FF input strength in the system via the self modification of network oscillation, this seems to be a very effective automatic gain control system that does not require any synaptic adaptation or learning mechanism [37].

Next, we examine how the oscillation activity affects the response delay of the network. Generally, the response time (time interval between an FF input spike and a corresponding output spike) is relatively long (∼10 ms) for weak inputs and becomes shorter as input strength increased for all cortical states (Figure 4B). But there is a significant difference in the average response time depending on the network activity state. Figure 4B shows that the average response delay is shorter during spontaneous oscillations than for the other two cortical activity states considered, especially when the FF input is weak. As input strength increases, the response of the oscillating network becomes similar to that of the oscillation inactive network, but still faster than that of the FF network. For a sufficiently strong FF input, the response time delay curve of the oscillating network and the oscillation depressed network were almost the same, with a delay of about 4 ms, agreeing with the experimentally known value for monosynaptic connections [35]. This is still faster than that measured for the FF network. Cortical interaction adds some positive current to each neuron, and this additional current causes the membrane voltage to reach the action potential threshold faster. In the presence of cortical oscillation, the amount of cortical current added is larger in the positive phase of the oscillation and on average, the larger the oscillation, the greater the average positive current that is added to the cell. That leads to faster responses than observed for the simple FF network. As the FF input strength increases above 35 µS/cm^2^, the oscillation amplitude decreases (Figure 5A), and the net average positive current added is less. The response time difference between oscillatory and FF networks decreases accordingly (Figure 5B). E-E cell interactions control the cortical oscillation strength, and the extent of response delay modulation and the response modulation of the network are in turn proportional to the cortical oscillation strength.

### Cortical Response Modification Is Localized: The FF Input Can Be Selectively Amplified in Particular Regions

**Figure 8:**
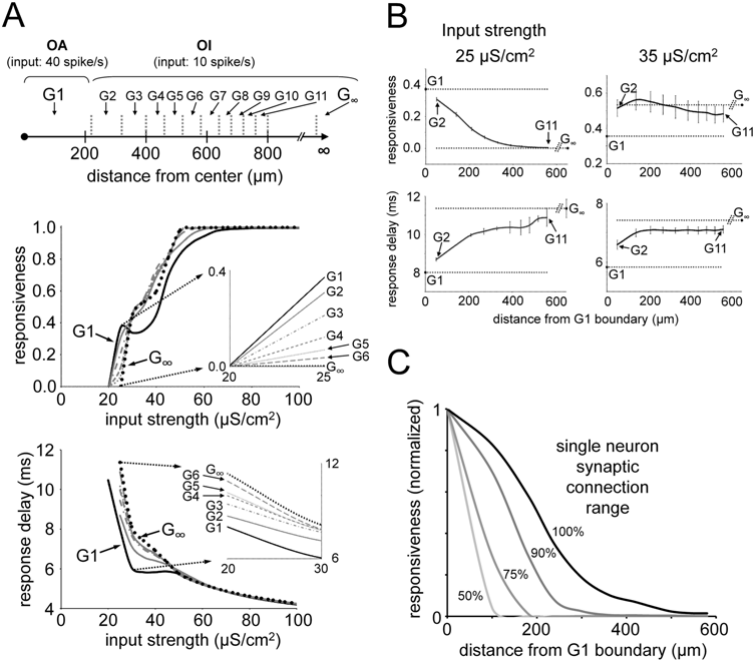
Localization of the oscillation activity effect in large cortical network (2 mm by 2 mm). (A) In the center region (G1), cortical gamma oscillation is activated by the higher FF input rate (40 spikes/s). Surrounding neurons are divided into groups (G2∼G11) by their distance from the center, and spontaneous oscillation is inactivated due to the lower FF input rate (10 spikes/s). The control group (G_∞_) property was separately achieved by a uniformly low FF input rate (10 spikes/s) network, as an approximation of infinitely distant neurons. Responsiveness and response delay measurements show that the properties of surrounding neurons (G2∼G6) are continuously distributed between G1 to G_∞_ (insets), and distant groups (G7∼G11) show almost the same property as G_∞_. (B) For FF inputs weaker than the FF response threshold (<30 µS/cm^2^), responsiveness and response delay changes gradually with the distance from the G1 boundary. The G2 property is very similar to that of G1, while G11 properties are almost the same as G_∞_ properties. For stronger inputs (>30 µS/cm^2^), surrounding regions are not much influenced by G1 oscillation. Most surrounding group properties are similar to those of G_∞_, showing clear localization of oscillation effect. For all FF input strength, the influence of oscillation is certainly restricted within local area. (C) The gamma oscillation effect localization is determined by the range of single neuron synaptic connections. The E- and I- synaptic connections of each single neuron are varied from their initial value (100%, radius of 200 µm for E- cells, 100 µm for I- cells), to 50% (100 µm for E- cells, 50 µm for I- cells). The ratio of E- to I- connections range (2∶1) was kept the same in all cases. The area of surrounding regions affected by the oscillation in the center region shrinks, as the E- and I- synaptic connection range is reduced. For comparisons, cortical responsiveness is normalized to the value of the center region in each case.

To examine the spatial localization of the cortical gain resulting from the spontaneous oscillation of network activity we used a network model four times the size (2 mm by 2 mm, 13000 neurons) of that used in the studies described above. In the center region (diameter∼450 µm, including ∼500 neurons, Figure 8A G1), the cells were activated as before with a FF input rate of 40 spikes/s, a rate that was also used to set up the spontaneous network activity oscillations for the simulations described above. The surrounding neurons (Figure 8A G2∼G11) received a signal with an FF input rate of 10 spikes/s, a rate at which spontaneous oscillations are hardly evident previously. The cortical neural connectivity is the same everywhere so that the center and the surrounding neurons could interact with each other. There are differences in the responsiveness of the network between the central region that shows strong spontaneous oscillations and the surrounding regions with little to no oscillations. In other words, the responsiveness modification by the spontaneous oscillation can be localized. A control simulation where all cortical neurons received an FF input of 10 spike/s in both the center and the surrounding regions is an approximation of the neuronal property at infinite distance from the center region (region G_∞_ in Figure 8A).

For measurement purposes the network is divided into circular annuli (Figure 8A G2∼G11). Each annulus contained 400 to 500 neurons. Figure 8A shows the network response function and the response delay time for single unpaired FF spike inputs in each region. Figure 8B shows the measured values in all the regions for FF input strengths of 25 and 35 µS/cm^2^ which are slightly smaller and slightly larger, respectively, than the FF response threshold value (S_thresh_ = 30 µS/cm^2^, Figure 4A). When the FF input strength was weaker than S_thresh_, the responsiveness and the response delay pattern gradually moved from the oscillation-active center region (G1) to approach the control oscillation suppressed case (G_∞_). Subsets of the Figure 8A graphs show that the quantities measured in the surrounding regions G2∼G6 are continuously distributed in the interval between the values obtained for G1 and G_∞_. Values measured in surrounding regions G7∼G11 showed negligible differences from those measured for G_∞_, and are not plotted in the figure. The effect of oscillatory network activity is restricted to a central local region about 500 µm in radius. When the FF input strength is larger than S_thresh_ but not very large (30 µS/cm^2^<S_input_<50 µS/cm^2^), the spatial attenuation of the cortical activity effect is more apparent (Figure 5B, S_input_ = 35 µS/cm^2^). In this case, all surrounding regions, even including the nearest region G2, are clearly separated from G1, and are close to G_∞_. In this case, the cortical oscillation activity effect is restricted to the central activated region (G1). For very strong FF inputs (50 µS/cm^2^<S_input_), all response properties converge to control group (G_∞_) behavior as expected since under that condition oscillatory behavior is barely evident even in the central region.

It seems reasonable to expect that the spatial localization of gamma oscillation is dependent upon the range of single neuron synaptic connectivity so that the shorter synaptic connection range, the smaller the surrounding area that is affected by the oscillation in the center. To verify this expectation, we reduced the excitatory and the inhibitory synaptic interaction range from the initial value (radius of 200 µm for E- cells, 100 µm for I- cells), keeping the ratio of E- and I- range the same. In Figure 8C, the range of oscillation effect is proportional to the single cell synaptic connection range, as expected. This suggests that the effective range of gamma oscillations is strongly dependent upon the details of the anatomical connectivity of neurons in experimental observations.

Thus the effect of cortical oscillation is fairly well localized for weak and moderate FF input strengths. Neural response properties are modified only within or near the area in which spontaneous oscillation is activated. This suggests that spatially selective cortical response modification is possible. Spontaneous cortical oscillations can be locally induced by spatially localized thalamic inputs, and the cortical response character can also be selectively tuned within a limited region.

## Discussion

As shown above, cortical responsiveness to a single unpaired FF input spike is enhanced by synchronized gamma frequency oscillations with the help of E-E neuronal interaction, particularly for weak FF input strengths. The cortical response modulation is proportional to the oscillation strength which is controlled by the network itself depending on FF input strengths. This cortical effect is spatially localized fairly tightly depending upon the range of cell connectivity, suggesting that each cortical area can be tuned selectively by well localized FF inputs. These findings are relevant to previous experimental and simulational results, and improve our understanding of the response character of the visual pathway.

### Gamma Oscillation with E-E Cells Interaction

It was previously thought that cortical E-E activity interaction is not essential for gamma rhythm generation but can modify the oscillation frequency and the phase of cell firing pattern [11],[28],[29]. A recent experimental study showed that gamma rhythms in E-E cells activity can change the E- and I- cells firing phase distribution, and suggested that it may suppress the gamma oscillation [25]. We have shown that recurrent E-E interaction significantly modulates the oscillation frequency, the firing phase distribution of E- and I- cells, and the oscillation strength, without extinguishing the cortical oscillation. Moderate levels of E-E interaction generally strengthen the oscillation, causing the ∼0-degrees E-I firing phase and the lower oscillation frequency (near beta range). As a result, it modulates the cortical response function that is clearly relevant to encoding/decoding of information. The fact that the effect of E-E interaction is self regulatory for FF input strength variation suggests a useful mechanism for the cortical gain control, without neuronal feedback loops from the visual cortex to earlier visual stages.

This suggests a general mechanism by which the same types of neurons in different cortical layers can have different properties due to the different coupling within each layer. In previous studies, it was reported that the upper and the lower layers of the cortex can have different oscillation characters and phase response properties [38],[39],[40]. Our observations about the phase and the frequency modulation of cortical oscillation by E-E coupling, suggest that different neuronal properties in different cortical layers may originate from the different lateral connectivity (especially E-E coupling) strength in each layer. For example, neurons in the different hippocampus regions (CA1 and CA3) show noticeably different firing phase distribution and correlation activity patterns in gamma oscillations [27]. Considering that the E-E couplings are significant in CA3 [41] while they are sparse in CA1 [42], this may be a situation in which the E-E coupling property plays an important role in the modulation of neural activity, as suggested above.

### Responsiveness Tuning with Fast and Slow Cortical Oscillations

Previous experimental work has shown that (i) the visual responsiveness of the cortical network is significantly enhanced by slow cortical oscillation [1] and (ii) the phase of slow theta rhythm (4∼8 Hz) oscillation modulates the high frequency gamma (80∼150 Hz) band oscillation power [22]. Here we provide a clue to a mechanism for modification of neural response properties by various types of synchronized cortical network activities. As shown above in the results section, when the gamma frequency oscillation is generated, it enhances the neuronal responsiveness to weak FF input. If this gamma power is modulated by slower (theta or lower frequency) rhythm, then the network responsiveness could depend on the phase of this slow oscillation. This suggests a simple and consistent basis for the modulation of high frequency oscillation power by lower frequency activity. Some previous experimental work has shown that the power and the frequency of gamma oscillations can be modulated by various factors such as the operation of fast spiking interneurons and some neuromodulators [10]. In our simulation, the strength of the gamma oscillations can be significantly modified by changing the strength or rate of the FF input, with the help of E-E interactions but without changing any individual neuronal properties or network connectivity features. In addition, our results on the gamma oscillation modulation mechanism do not require modifying the FF input correlation pattern, learning/adaptation behaviors [37] in cortical synapses, or cortico-thalamic feedbacks [43]. If the slow frequency oscillations affect the FF input strength or the input rate locally within the network, the gamma oscillations will be readily modulated. The consequent modulation of the responsiveness will depend on the phase of the low frequency oscillation [1].

### Controlled and Selective Responsiveness Modification

This proposed responsiveness tuning mechanism does not require any accompanied background activity control. Therefore it is simpler than those gain control models suggested in the previous reports [2],[3],[4] that are mostly dependent on the modulation of the background activity. An important advantage of the present model is that the response modification can be ‘dynamically selective’ according to the FF input strength and the FF input projection range variation. Since the cortical gain is modulated by FF input strength, the cortical amplification is selective to FF input strength. The system effectively determines the ‘optimized’ gain via modulations of oscillation power spontaneously, and can avoid unnecessary adjustments even without any delayed feedbacks to thalamus or thalamo-cortical neurons. Moreover, the tuning is spatially localized to distances of less than about 500 µm for weak FF inputs, and less than 50 µm for strong inputs. This is comparable or even smaller than the size of the receptive field of single neuron in the mammalian primary visual cortex [44]. Therefore responsiveness modulation can be spatially selective, and this is more effective than mechanisms proposed in previous studies [2],[3],[4] where the cortical modulations were generally global and the gain optimization could not be achieved easily. We also suggest that this mechanism might be applicable to the functional modulation of the relative weight between thalamic inputs versus cortical inputs to the visual cortex neurons [45].

### Attended or Awake State Animal Behavior with Oscillations

In some experiments with monkeys, when attention is directed, visual sensitivity increased due to the increased synchronization between the visually evoked potentials and the stimulus [46]. In another report, neurons activated by the attended stimulus showed increased gamma frequency synchronization [47]. Considering the response enhancement by gamma oscillation in our model together with these experimental measurements, spontaneous gamma band activity seems to play an important role for regulating the information flow in the visual nervous system, as suggested previously [6],[16]. Our findings support these experimental observations, and may suggest a new mechanism for attention modulation that is different from that of other models [48],[49],[50].

## Methods

### Cortical Neural Network Modeling

This neural network model consists of a two-dimensional layer of coupled neurons, each modeled as a Hodgkin-Huxley neuron with Na^+^, K^+^ and Cl^−^ ion channels and E- and I- synaptic conductance channels. 75% of the neurons are E- and 25% are I- as suggested by experimental data [51], and adopted in other publications [24]. We used networks of two sizes in this research: (i) 1 mm by 1 mm, including ∼3300 neurons for responsiveness simulation and (ii) 2 mm by 2 mm, including ∼13400 neurons for locality simulations.

The membrane potential of the j^th^ neuron, 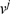, is determined by
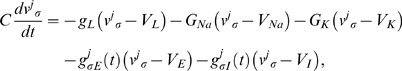
where σ is E or I depending upon whether the neuron is E- or I-, respectively, C is the membrane capacitance, and g_L_ is the leakage conductance. g^j^_σE_ is the synaptic conductance of the j^th^ neuron, E- or I- as specified by σ, providing the cortical inputs from the neurons in its spatial neighborhood, and g^j^_σI_ is the synaptic conductance of the j^th^ neuron providing the I- input from the neurons in its spatial neighborhood. We used the commonly accepted biophysical parameters [52],[53]: the capacitance C = 10^−6^ Fcm^−2^, the leakage reversal potential V_L_ = −70 mV, the Na^+^ equilibrium potential V_Na_ = 55 mV, the K^+^ equilibrium potential V_K_ = −80 mV, the E- reversal potential V_exc_ = 0 mV, the I- reversal potential V_inh_ = −80 mV, the leakage conductance g_L_ = 50*10^−6^ Scm^−2^, and Hodgkin-Huxley Na^+^ and K^+^ conductances g_Na_ = 120*10^−3^ Scm^−2^, g_K_ = 36 *10^−3^ Scm^−2^.

The Hodgkin-Huxley ion channel conductance takes the form [54]:
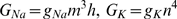
where m, h and n denote the channel activation variables.
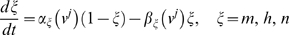



The rate constants 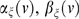 take empirically known forms [55]:
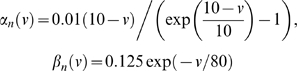


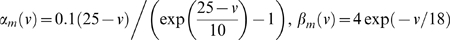


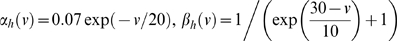



A j^th^ neuron's synaptic conductance is given by:
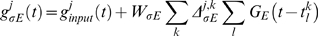


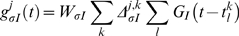




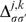
, 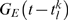 and 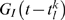 are the spatial, the E- temporal, and the I- temporal kernels of cortical interaction, respectively, which describe the contribution of l^th^ spike from k^th^ neuron to the j^th^ neuron.

For cortico-cortical connection, the spatial kernel in the synaptic conductance equation takes the form:
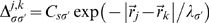
where 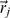 and 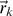 are the j^th^ and k^th^ neurons' spatial positions respectively. The decay constant, 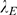 is 200 µm (for E- connections) and 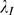 is 100 µm (for I-). The temporal kernel in the equation is set to be
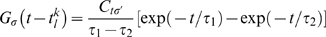
and the time constants 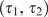 in milliseconds were chosen as (3, 1) for E- and (7, 1) for I- synapses where C_sσ′_ and C_tσ′_ are normalization constants chosen so that that the sum of the contributions of the two kernels would sum to unity.

We assume spatially isotropic local connections with a range of 200 µm in radius for E- and 100 µm in radius for I- synapses. W_σσ′_ are strengths of synaptic connections for the neuron pair of type (σ, σ′). If all W_σσ′_ = 0, the network is then equivalent to the simple FF model. When the cortical synaptic connections were turned on, these values ratios were set to (W_EE_, W_IE_, W_EI_, W_II_) = (0.03: 0.06: 0.12: 0.12) throughout the simulation. This condition was achieved from the parameter search simulations shown in the first part of results section.

The contribution to the E- conductance by the FF input spikes was given by:
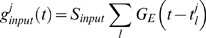



S_input_ is the weighting factor for FF input synaptic strength and *g^j^_input_* was varied within 5∼100 µS/cm^2^, throughout the simulations reported here. The temporal kernel 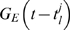 has the same form as the E- temporal cortical kernel given above. The spike timings, 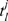, of input were generated by Poisson processes.

### Computer Simulation and Data Analysis Techniques

All of our simulations were coded using the GENESIS 2.3 environment [55], and performed with a Pentium IV PC system. Simulation outputs were analyzed using Matlab R2006b scripts.
